# Effects of the COVID-19 Outbreak on Elder Mistreatment and Response in New York City: Initial Lessons

**DOI:** 10.1177/0733464820924853

**Published:** 2020-05-08

**Authors:** Alyssa Elman, Risa Breckman, Sunday Clark, Elaine Gottesman, Lisa Rachmuth, Margaret Reiff, Jean Callahan, Laura A. Russell, Maureen Curtis, Joy Solomon, Deirdre Lok, Jo Anne Sirey, Mark S. Lachs, Sara Czaja, Karl Pillemer, Tony Rosen

**Affiliations:** 1 Weill Cornell Medicine/NewYork-Presbyterian Hospital, Department of Emergency Medicine, New York, NY, USA; 2 Weill Cornell Medicine/NewYork-Presbyterian Hospital, Division of Geriatrics and Palliative Medicine, New York, NY, USA; 3 The Legal Aid Society, New York, NY, USA; 4 The Legal Aid Society, Bronx, NY, USA; 5 Criminal Justice Programs, Safe Horizon, New York, NY, USA; 6 The Harry and Jeannette Weinberg Center for Elder Justice at the Hebrew Home at Riverdale, Riverdale, NY, USA; 7 Weill Cornell Medicine/NewYork-Presbyterian Hospital, Department of Psychiatry, New York, NY, USA; 8 College of Human Ecology, Cornell University, Ithaca, NY, USA

**Keywords:** elder mistreatment, elder abuse, COVID-19, intervention

## Abstract

New York City is currently experiencing an outbreak of COVID-19, a highly contagious and potentially deadly virus, which is particularly dangerous for older adults. This pandemic has led to public health policies including social distancing and stay-at-home orders. We explore here the impact of this unique crisis on victims of elder mistreatment and people at risk of victimization. The COVID-19 outbreak has also had a profound impact on the organizations from many sectors that typically respond to protect and serve victims of elder mistreatment. We examine this impact and describe creative solutions developed by these organizations and initial lessons learned in New York City to help inform other communities facing this pandemic and provide guidance for future crises.

## Introduction

Coronavirus disease 2019 (COVID-19) is a novel infectious respiratory virus that was first discovered in Wuhan, China and has since become a global pandemic, with cases currently in more than 185 countries (Coronavirus Resource Center, Johns Hopkins University & Medicine, 2020). The pandemic already has had staggering medical, social, and economic consequences, with more than 156,064 deaths as of April 18, 2020 (Coronavirus Resource Center, Johns Hopkins University & Medicine, 2020), health care systems stretched and discussing how to ration care if necessary, and large populations confined to their homes. The cost of the pandemic could reach $4.1 trillion (Alegado, 2020), and countries around the world are moving toward recession. The scope, size, and severity of this global public health disaster is unlike any seen in the last 100 years. In addition, the virus is not yet under control, with hand washing and social distancing among the only preventive measures and supportive care the primary treatment. Most regions of the world are seeing their first cases and bracing for anticipated future outbreaks.

Older adults are particularly susceptible to the impact of COVID-19, with more severe illness and higher mortality among adults aged ≥65 and those with co-morbid conditions including diabetes and cardiovascular disease, which are more common in this population. Currently, eight out of 10 COVID-19-related deaths reported in the United States are older adults (Centers for Disease Control and Prevention, 2020). Furthermore, this disease is highly contagious, putting those who live in close quarters at increased risk. The impact of social distancing is also especially problematic for older adults, as many already confront problems with social isolation and loneliness. Social distancing requirements limit access to medical care, interactions with family and friends, and access to social support structures.

Increased attention is urgently needed for a particularly vulnerable group of older adults: victims of elder mistreatment and those at risk of victimization. Elder mistreatment is common, affecting as many as 10% of community-dwelling older adults each year (Pillemer et al., 2016), with nursing home residents also at risk. This mistreatment may include physical abuse, sexual abuse, neglect, verbal/emotional/psychological abuse, and financial exploitation, with many older adults suffering from multiple types concurrently.

COVID-19, a unique crisis, has also had a profound impact on the organizations from many sectors that typically respond to protect and serve victims of elder mistreatment. In this commentary, we explore the impact and describe creative solutions developed by these organizations as well as initial lessons learned in New York City, which is the epicenter of the outbreak in the United States. We hope this information will help inform other communities facing this pandemic and provide guidance for future crises.

## Impact on Elder Mistreatment Victims

Older adults are disproportionately affected and more likely to die in nearly all natural disasters, as evidenced by recent floods, heat waves, earthquakes, hurricanes, and tsunamis (Gutman & Yon, 2014). Family violence, including child abuse (Curtis et al., 2000) and intimate partner violence among younger adults (Parkinson & Zara, 2013), has also been shown to increase in the aftermath of these disasters. Although very little literature exists examining the impact of natural disasters on elder abuse, the Illinois Department of Aging reported that areas affected by a major flood in 1993 experienced a 38% increase in the reports of elder abuse, neglect, and exploitation during the year following the flood compared to the previous year (Oriol & Nordboe, 1999). In addition, some elder mistreatment is intimate partner violence that has continued into older age. In most natural disasters, many of the issues involve displacement from living environments, movement to shelters, and then repairing/rebuilding and return. As a result, much of the elder mistreatment surrounding disasters is described anecdotally and, in the limited literature available, has included abandonment of functionally or cognitively impaired older adults unable to leave nursing homes or other living environments, neglect and theft while in shelters, and fraud by contractors and others overseeing repairs/rebuilding and return (Gutman & Yon, 2014).

COVID-19 and the response to the virus represent a very different type of disaster with very different potential consequences, including increasing risk factors of elder mistreatment. Rather than displacing older adults from their living environments, shelter-in-place orders confine them there. The consequences of this restriction for victims of elder mistreatment, though not yet systematically researched, are potentially devastating. In addition, places of gathering, such as senior centers and houses of worship, are no longer open, resulting in less access to the community, increased social isolation, and fewer opportunities for informal surveillance. Social isolation itself is a well-known risk factor for elder mistreatment (Pillemer et al., 2016).

Furthermore, some home health aides (HHAs) may be unable to continue to care for older adults due to illness or agency policy. Other HHAs may be unwilling to provide care due to fear of contracting COVID-19 either in traveling to and from the older adult’s home or in providing care. In some instances, families may choose to cease care from HHAs to reduce possible exposure. This places greater strain on family and informal caregivers, who may already be suffering from economic or psychological trauma due to the pandemic. As a result, family could be more prone to become abusive or neglectful, or to escalate existing mistreatment, due to challenging life circumstances and caregiver stress. Furthermore, the absence of an HHA eliminates the possibility of a witness to potentially abusive or neglectful behavior, reducing the ability to prevent or detect mistreatment.

Older adults already experiencing mistreatment at home, or living with family members during this time they otherwise might not be cohabitating with, may be put at higher risk for mistreatment while simultaneously making detection more difficult. For example, an abused, neglected, or exploited older adult may be in a confined space with the abuser and unable to reach out to friends, family, or community supports in a safe manner. Neighbors, friends, or others who typically informally check on older adults may also be reluctant to visit given the risk of exposure to the virus, both for themselves and the older adult, causing further isolation.

Evidence is already emerging from other countries that stay-at-home orders are increasing intimate partner violence in younger adults, as families are forced to spend more time together (Taub, 2020). In Spain, during the first 2 weeks of lockdown, the emergency number for domestic violence received 18% more calls than in the same period a month earlier, and French police recently reported a nationwide spike of 30% in domestic violence (Taub, 2020). A similar pattern is likely in elder mistreatment, but older adults, particularly those with functional and cognitive impairment, will have more difficulty accessing help or not know how to do so.

Furthermore, COVID-19 has created enormous economic uncertainty, with a significant number of workers laid off or furloughed. As many older adults have savings and receive stable monthly incomes, unemployed family members and others may be tempted to inappropriately access and use these funds. In addition, the US government has announced payments to individuals to offset anticipated financial losses associated with COVID-19, which may also be misappropriated by an abuser. As noted by The U.S. Department of Justice (2020), several ongoing scams are attempting to exploit individuals during this crisis, such as malicious websites and applications that appear to share fraudulent virus information to gain access to devices. Other scams are attempting to sell fake cures, seek donations for illegitimate or non-existent charitable organizations, and send phishing emails imitating organizations such as the World Health Organization (WHO). During this time, some older adults will become more susceptible to such scams and exploitation.

## COVID-19 in New York City

The first case of COVID-19 in New York City was discovered on March 1, 2020 (Goldstein & McKinley, 2020). Initially, public health strategies included tracing contacts of infected persons and quarantining those with known exposure. However, it soon became clear that community spread within New York City was pervasive, and public officials put in place a series of measures, similar to those already implemented in communities in other countries, intended to increase social distancing and slow viral spread to avoid overwhelming the local health care system. Schools and restaurants/bars closed on March 16, 2020, and a stay-at-home order was issued on March 20, 2020. Despite this, New York City quickly became the epicenter of the outbreak in the United States. As of April 17, 2020, 122,148 cases have been diagnosed, even in the absence of widespread testing, and there were 7,890 deaths (73% aged ≥65)—on the most deadly day, 509 New Yorkers lost their lives (New York City Department of Health, 2020). Many health systems and hospitals have been stretched beyond their capacity, with the city seeking state and federal assistance to provide care to patients.

## Impact on Organizations in New York City That Respond to Elder Mistreatment

The COVID-19 pandemic and New York City’s strategies to manage spread have had a significant impact on the organizations within the city that respond to elder mistreatment. Adult Protective Services (APS), of the New York City Human Resources Administration, the primary organization responsible for investigating potential elder mistreatment cases and responding to concerns for at-risk older adults, has had to modify its processes both internally and in the community. On March 23, 2020, personnel transitioned to working remotely. This shift was an enormous challenge, requiring a tremendous amount of IT support to negotiate the issuance of tablets, smart phones, and laptops. Staff also had to obtain remote access to sustain operations. Within this transition was the conversion of the largest APS rep-payee program (a program in which APS is appointed by the Social Security Administration to manage clients’ social security benefits) to a remote operation that would issue 5,000 checks without delay.

During this time, APS caseworkers have been encouraged to initiate contact remotely with at-risk individuals, caregivers, or collateral contacts prior to conducting a home visit to obtain health information and assess safety. Personnel are utilizing a variety of video-conferencing tools such as Facetime, Skype, and other technological resources, to facilitate remote assessment when possible. However, such remote work can be challenging, as it may not be safe for victims to speak on the phone if they reside with the actual or potential perpetrator. The abuser may even prevent the older adult from having access to this technology. In addition, some older adults do not have cell phones or have limited ability to communicate due to cognitive or physical impairments. As a result, home visits continue to be necessary to determine risk, eligibility, and the provision of appropriate services.

APS also modified its process for conducting home visits, the cornerstone of their investigation and intervention approach. Attention needed to be focused on cautionary measures and providing guidance to caseworkers who visit homes at which an older adult, or anyone with whom they live, is exhibiting symptoms of COVID-19. When making such visits, it is essential that caseworkers have proper personal protective equipment (PPE) to safeguard both the client and the APS worker, thus reducing opportunity for exposure. Per a New York City Mayoral directive issued April 13, 2020, all city public employees were required to wear masks and gloves when interacting with the public they serve to minimize possibility of virus transmission. APS benefited from a shipment of PPE delivered April 10, 2020, providing staff with a significant supply of masks, gloves, and hand sanitizer, and expects to continue to receive PPE materials for frontline staff as the health crisis continues.

Although APS continues to engage partners with whom they typically collaborate, they recognize that such agencies are overburdened and may not provide as robust a response. Given the need to redeploy resources, the New York City Police Department (NYPD) is more limited in its ability to conduct wellness checks on older adults. However, they are still able to conduct callbacks, review 911 calls, and collaborate with community partners. APS has been able to establish close relationships with individual Domestic Violence Police Officers (DVPOs), specialized police officers who work with domestic violence survivors, allowing for discussions to determine if a DVPO visit is necessary. In addition, with the city’s Emergency Medical Services (EMS) already stretched thin, APS has been consulting with community partners to explore all available options before calling 911. Concerns for hospitalization during this time are amplified because if elder mistreatment victims are brought to the Emergency Department (ED), they may be exposed to the virus. Furthermore, if there is no medical need for admission, at-risk older adults may be discharged back home to the unsafe environment as the capacity for hospitals to admit and manage these complex social situations dwindles. Hospital/ED-based elder abuse intervention programs, such as the Vulnerable Elder Protection Team (VEPT) at New York Presbyterian/Weill Cornell Medical Center (Rosen et al., 2018), have had to scale back the services they provide and redeploy medical providers who work within the program to provide care for COVID-19 patients. To counter the effects of such changes, APS has employed their staff nurses and social workers to facilitate linkages to client’s physicians/medical network in an effort to avoid hospitalizations.

Other community agencies are facing their own challenges. The closure of senior centers has reduced opportunities for detection and reporting of mistreatment-related concerns. In addition, it has made it more difficult for older adults to access other forms of support such as socialization, independent nutritional support, and credible information and health protection guidelines. As a result, city agencies are forced to respond in creative ways to ensure that older adults are properly served. Community-based counseling and civil legal programs are no longer offering in-person services, now providing counseling over the phone. Of course, phone-based services are challenging for older adults who are sheltering-in-place with an abuser and who also may have more difficulty using telephonic communications due to high rates of hearing impairment and language barriers for those with limited English proficiency. Ombudsman programs are prevented from entering facilities during the crisis due to the Center for Medicare and Medicaid Services (CMS) ban on visitations. As a result, they must rely on phone or video chat to connect with residents. Unless a resident knows how to call the Ombudsman for help, their access to such services is extremely curtailed.

The new circumstances have also affected the city’s network of multidisciplinary elder abuse teams. Weill Cornell Medicine’s New York City Elder Abuse Center (NYCEAC) is the organization responsible for coordinating the enhanced multidisciplinary team (EMDT) meetings in each borough of New York City, convening representatives from medicine, aging, elder abuse, law enforcement, criminal justice, civil legal, forensic accounting, protective services, guardians, and community-based organizations to regularly discuss and collaborate on challenging elder abuse cases. While EMDTs are traditionally held in-person, NYCEAC shifted exclusively to virtual meetings even prior to the shelter-in-place order, without any disruption to the schedule. Understanding that agencies may have to redeploy resources during this health crisis, remote EMDTs have allowed agencies, such as APS and DVPOs, to remain in close dialogue, which is critical to safety planning with clients.

Among the greatest challenges for all organizations serving victims of elder mistreatment has been the frequent changes in the guidance from public health and other officials on appropriate social contacts. As a result, many organizational leaders found themselves continually building and rebuilding protocols to optimally and safely protect and serve older adults while preventing exposure to workers. This poses unique challenges for NYC given the diversity of the five boroughs. Other communities should anticipate these profound impacts on service delivery and begin preparing for them as soon as possible.

## Creative Solutions

New York City organizations that serve vulnerable older adults have already developed creative and innovative solutions (Table 1) to respond to this unprecedented crisis, which may be useful to other communities.

**Table 1.** Summary of Creative Solutions Developed in New York City and Considerations for Other Communities in Potentially Adopting Them.

NYC strategy	Considerations
Weekly telephone meetings including leaders in the local elder abuse community from various agencies and backgrounds	• Ideal if organizations already have existing working relationships (but this should not deter communities from beginning to develop infrastructure) • Facilitator/organizer needed who can coordinate schedules/set up meetings • If community has an elder abuse Multidisciplinary Team, this infrastructure may be useful in developing plan and roster for these leadership meetings
Transition to remote work seamlessly	• Guidelines and policies are needed for staff to work effectively remotely • Remote access to critical databases and information for key personnel should be obtained, and procedures for remote access developed • Tablets, smart phones, and laptops should be obtained/distributed as necessary • Plans should be developed for large operations, such as rep-payee programs, to be converted to operate remotely • Staff call-trees can be created for use in an emergency to facilitate agency communication
Modifications to APS referral system to maximize capture of important information at initial intake, including information about COVID-19	• Modified intake process to be developed and deployed • Communication with staff and partners, as well as the community, about modified intake process is critical • Must ensure staff available to support new intake process, including responding to phone/e-mail referrals if appropriate
Continuing to conduct investigations and serve APS clients while limiting risk to caseworkers	• Guidelines are needed to ensure that APS caseworkers are not putting themselves at risk unnecessarily while recognizing the importance of investigations as part of APS’ work • Contact should be attempted before a home visit to obtain health information and assess safety • Video-conferencing tools such as Facetime, Skype, and other technological resources should be utilized to facilitate remote assessment when possible • Personal protective equipment for APS should be obtained as soon as possible, and caseworkers should be trained on its use to ensure safety of workers and clients • Strategies and protocols for check in/follow-up via telephone and use of neighbors/family for additional surveillance should be put into place when in-person investigations are not possible • Formally track clients and caseworkers who may have been exposed to COVID-19
Maintain elder abuse Multidisciplinary Team meetings, even if remotely	• Effective safety planning for complex cases requires collaboration and understanding the changing capabilities of different organizations even more than under normal circumstances
Changes to police department domestic violence response strategies	• Collaboration with APS and other organizations may assist with identified prioritizing highest risk older adults and families • Personal protective equipment for police officers should be obtained if possible, and police should be trained on its use
Reach out to older adults typically served by senior centers and focus on providing food and delivering other services remotely	• Senior centers should consider contacting participants regularly • Opportunities to deliver other services remotely should be explored • Food delivery programs should be developed and scaled quickly
Changes to court system allowing for remote processes and added protections during this uncertain time	• Capability to process order of protection extensions quickly and remotely should be developed and/or expanded • Technology and protocols to allow more cases to be heard virtually, such as those for new Orders of Protection • Ability to conduct more guardianship hearings and hearings on related issues remotely • Plan for or expand upon potential to hear urgent COVID-19-related applications
Support for frontline staff under this additional stress	• Regular meetings, check-ins with front line staff • Social work staff already working in agencies or others who may have training in crisis counseling may be able to assist with this role • Access to mental health resources, peer support • Consideration of changing schedules/making more flexible to allow workers more time with family
Consider strategies to support older adults and reduce stress on social services during this time	• Helplines/hotlines should be launched or expanded to allow older adults and concerned persons to obtain information and navigate new processes • Consider expansion given the amount of advice/support to older adults and concerned persons that will need to be delivered remotely during this period • Develop a schedule for regular remote check-ins with at-risk clients • Consider conducting elder abuse survivor support groups remotely • Create processes to deliver meals to seniors at home and recognize other needed services that aren’t being received • Food stamps and other resources should be made available online • Delay evictions and foreclosures during time of upheaval, though recognize this may make it more difficult to evict potential perpetrators

APS = Adult Protective Services.

### Transitioning to Remote Meetings

Recognizing that recommendations from public officials were constantly changing and that capabilities of individual agencies were always in flux, NYCEAC began to facilitate weekly phone meetings, including leaders in the local elder abuse community from various backgrounds, such as APS, Jewish Association Serving the Aging (JASA), New York City Department for the Aging, NYPD, the Mayor’s Office to End Domestic and Gender-Based Violence, the New York City Sheriff from the Department of Finance, Legal Aid, the New York State Judicial Committee on Elder Justice, Weinberg Center for Elder Justice, Safe Horizon, and VEPT. These remote meetings have already become indispensable and help keep providers aware of service disruptions, new guidelines, and other obstacles, while also helping to brainstorm novel solutions. Opportunities for service collaborations and ways to manage particularly challenging issues have emerged as well. In addition, these meetings serve as an important way to help professionals cope with the challenges they face and provide emotional support, reassurance, and advice about leading organizations with frightened workers serving vulnerable clients during this unprecedented time. Continuing EMDT meetings remotely in each borough without any pause or cancellation has served similar purposes.

In addition, the Mayor’s Office to End Domestic and Gender-Based Violence holds bi-weekly telephone meetings for all city agency and community-based organization stakeholders. Over 100 people call-in to receive up-to-date information about the city’s response to COVID-19 as it pertains to domestic violence. People can also ask questions through the chat feature to the Commissioner, Assistant Commissioners, and others during the meeting. Bulletins are emailed the next day summarizing the shared information. HRA also hosts weekly meetings for domestic violence shelter providers to address issues arising in the City’s domestic violence shelter system and share updates on policy and programmatic changes.

### Modifications to APS Procedure and Support for Staff

APS has also innovated its approach to serving vulnerable older adult clients. They have modified their intake process and protocol, suspending the central online form-based intake process that had been available. Taking referrals via phone or e-mail provides an opportunity to obtain additional case details, which have helped caseworkers and APS leadership prioritize cases, know in advance the level of risk of COVID-19 exposure, and make plans for visits to be as efficient and effective as possible. Psychiatrists who work for the city have been using telemedicine to evaluate APS clients, when possible. If appropriate, Visiting Psychiatrists Services (VPS) has also continued to do home visits along with their APS counterparts, using a triaging process to not overwhelm the partnership.

DVPOs suggested that APS capitalize on New Yorkers who are sheltering-in-place by seeking assistance from neighbors who might be able to offer critical information about the circumstances in the older adults’ home. These individuals might be able to encourage victims to reach out to friends and family by using the guise of a pandemic as “a good time to connect with people,” to decrease isolation and attempt to ensure safety. In addition, DVPOs can refer older adults to a Safe Horizon Crime Victim Assistance Program (CVAP) Advocate, who work in police precincts, to provide an enhanced safety and needs assessment remotely and facilitate connection to other services as needed.

Notably, APS has developed a document to track COVID-19-related interactions to help staff identify clients who may require additional follow-up and support during this health crisis. Another form was created to track staff and clients who have been exposed to COVID-19 to help minimize risk of transmission and help ensure follow-up and safety. Both documents are in an application available with Office 365 and are only accessible by managers.

To support its own personnel, APS leadership has dedicated time to reach out to caseworkers on the front lines to offer support and a safe space to express fears and concerns. APS has also enlisted their social work supervisors to call staff while they telework from home, to see how they are coping during this crisis, how are they caring for themselves, and how the administration can further support them. Similarly, recognizing the toll of this “new normal,” Safe Horizon is providing support to staff, both individually and as a group, by offering trauma experts from within and outside the agency and providing “bonus pay” to personnel unable to work remotely. They also address the disproportionate impact that COVID-19 is having on communities of color, by fostering conversations about racial trauma with consultants from racial equity organizations, because many of the Safe Horizon frontline workers are impacted professionally and personally by this stark reality.

### Innovative Law Enforcement Response

NYPD has been reaching out via phone to known victims of elder mistreatment or domestic violence to help safety plan and implement code words (Parascandola et al., 2020). DVPOs are also able to link 911 calls to specific locations, in instances when individuals do not or cannot answer the phone, and conduct a home visit. In addition, Safe Horizon CVAP and Court Advocates, who are now working remotely, have provided follow-up calls to older adult clients to conduct additional safety and needs assessments and develop a safety plan that addresses their unique needs. Calls may include exploring how neighbors or family could assist, discussing the terms of an order of protection, a referral to the Safe Horizon 24/7 Hotline or Safechat Program, connection to the Safe Horizon Project Safe Program to have house locks changed for free, and linkage to the NYC HRA Alternative to Shelter Program, a program that installs a security alarm in the home free of charge.

### Addressing Meal Delivery

To ensure the basic needs of older adults are met during the COVID-19 crisis, the city instituted an emergency food program to facilitate meal delivery. Taxis have been deployed to deliver food to older adults, free of charge. Senior centers are calling all participants regularly to check on them and ensure they are receiving meals. Public benefits, such as food stamps, are now accessible online and by phone, with essential HRA staff prepared to provide assistance to anyone who needs help managing their case or applying by phone. The city has obtained federal waivers so that benefits can be granted without meeting all of the previously required criteria, such as a face-to-face interview.

### Responding to Housing Concerns

To address housing concerns, which are often present in cases of mistreatment, HRA has established a housing helpline staffed by legal service providers cooperatively to provide support. By expanding the inclusion criteria, they are able to serve a wider array of individuals. Evictions and foreclosures have also been suspended for 90 days, which provides a much-needed safety net for at-risk older adults but can also make it more challenging to evict potential abusers.

### Involvement of the Courts and Legal System

New York State has instituted virtual court parts for emergency matters, which includes allowing individuals of any age to petition remotely for a new family court order of protection with the help of Safe Horizon’s Family Court Programs, advocates and attorneys working out of the City’s Family Justice Centers, and other community-based organizations. In addition, staff discuss with the older adult the process of serving and enforcing the order and conduct safety planning.

The Supreme Court’s Civil Branch is remotely hearing applications for temporary orders of protection in matrimonial matters. Supreme civil, family, and criminal courts are automatically extending temporary orders of protection. Urgent cases with imminent risk requiring a temporary guardian are being heard remotely. Guardianship judges in New York County Supreme Court, who are working remotely on a rotating basis, are also hearing other applications, such as issues surrounding when someone subject to a guardianship is being released from the hospital or care facility. Issues raised have included ensuring that an appropriate after-care plan is in place, including housing, medications, food, and other needs. Courts have also begun hearing essential applications related to COVID-19. For example, an 81-year-old woman with a number of health issues, fearing exposure to the virus, sought exclusive use and occupancy of the marital residence after her husband had been out of the home since December and wanted to return with his HHAs.

The Legal Aid Society is operating remotely for all civil legal questions. Calls are routed through a central call system that distributes them in real time to staff members who can gather appropriate information and offer immediate advice. Attorneys may also take cases for full representation with a very quick turnaround.

### Helpline

NYCEAC’s Elder Abuse Helpline for Concerned Persons (Elder Abuse Helpline) supports concerned persons, including family members, friends, or neighbors who are impacted by and involved in the lives of elder abuse victims. Given the nature of its service (phone and internet-based), Elder Abuse Helpline operations have not been impacted by the pandemic, but calls have increased during this period, with many involving conversations about problem-solving related to minimizing risk of COVID-19 exposure, while also keeping older victims safe from mistreatment.

### Shelters

Domestic violence shelters remain an option for those who need to flee their home, and Domestic Violence Hotline staff are available 24/7 to help older adults explore this option. The Weinberg Center for Elder Justice, a shelter for elder abuse victims located at the Hebrew Home at Riverdale, has also continued to accept admissions. In addition to existing admission protocols, the chief medical officer now reviews any potential admission as an added precaution of medical safety.

In addition, the Weinberg Center leads the SPRiNG Alliance, spring-alliance.org, a professional network of elder abuse shelters with members across the country, which has continued its monthly calls and added an additional COVID-19 monthly check-in. Shelter partners in the SPRiNG Alliance continue to benefit from sharing current challenges and innovative solutions, including safety planning at home and remote court appearances.

### Mental Health

Weill Cornell’s PROTECT program delivers mental health services to elder abuse victims with depression or anxiety (Sirey, Berman, et al., 2015; Sirey, Halkett, et al., 2015). The program, which previously made in-home visits or met clients in local safe locations such as libraries, senior centers, or parks, has switched to telephone or video services. This shift enables clinicians to continue to work with the victim, but is often fraught with new issues such as privacy and confidentiality, limited technology expertise, and the absence of the benefit of getting out of the home. However, the PROTECT program continues to receive referrals and provide treatment in whatever modality is best for the victim in both English and Spanish city-wide.

### Telephone and Remote Monitoring

Department for the Aging (DFTA)-funded community based elder abuse programs are maintaining and expanding services through remote visits with clients. JASA’s Legal Social Work Elder Abuse Prevention Program, a large, unique agency providing comprehensive services to many at-risk clients, has instituted a weekly phone contact protocol to monitor each client’s status, update safety planning, coordinate existing services, and address emerging needs. JASA attorneys and social workers continue to coordinate and represent clients remotely, working to secure emergency orders of protection and prevent mortgage fraud by electronically submitting petitions to the courts. In an effort to preserve social connectedness while addressing physical distancing, JASA has replaced its regular in-person groups with weekly telephonic support groups for elder abuse survivors. Led by a JASA social worker, these groups utilize a mutual aid model, providing crucial peer support, information, and practical guidance for vulnerable older adults who are experiencing increased challenges related to the health crisis. JASA’s community health nurse participates in selected group sessions, providing health information and instruction for managing stress, accessing telehealth services, and managing physical distancing and hygiene recommendations.

We hope that some of these creative solutions might prove useful to communities who may soon be affected by this challenging pandemic.

## Lessons Learned for Communities to Consider

As organizations in New York City continue to work together to optimally serve elder mistreatment victims during this pandemic, we have also learned lessons that may inform communities facing this crisis in the future. We recognize that community resources vary across localities, as does internet access, so some of these lessons may not be transferrable. We strongly recommend, however, that cities and towns across the country consider the following options in particular:

1. *It is imperative to convene regular meetings with professionals responding to elder mistreatment cases as soon as possible, regardless of whether COVID-19 has reached your community.* Preparation for a possible crisis is critical to continuing to provide services. Furthermore, should COVID-19 emerge, regular and frequent meetings to assess and share current practices should be held. Multidisciplinary Teams may help facilitate these conversations, but certainly are not required. Organizations have reported using Google docs or other shared platforms to keep staff updated on service changes as they occur.
2. *The ability to visit a home is a key component of an APS investigation and, as a result, is a vital part of ensuring at-risk older adults are protected*. APS is able to safely perform this function with access to and training on how to appropriately use PPE. Although there is a PPE shortage in New York City, and limited supplies should certainly be given to frontline health care workers, inadequate access for APS personnel leaves caseworkers and leadership with the agonizing decision of whether to visit an older adult in crisis and risk exposure or not fully investigate the situation and have limited ability to intervene. Therefore, communities should try to ensure that APS is equipped with PPE before the outbreak. Fortunately, the U.S. Department of Homeland Security recently identified APS as first responders. Hopefully, this designation will strengthen state and local APS offices’ advocacy efforts when working to secure PPE for their staff.
3. *APS and community service workers must be confident in how to advise vulnerable older adults to protect themselves from illness exposure through social distancing and hand washing*. In many homes where vulnerable older adults live, other family (including potentially the abuser) may have COVID-19. In addition, if those infected are also the primary caregivers of the older adult, plans for substitute care need to be implemented quickly. Ideally, APS would also have options to remove vulnerable older adults from the home if necessary.
4. *Home health agencies and nursing homes should educate their workforce on how to protect themselves from COVID-19 and ensure appropriate PPE is provided*. Aides should be educated on how to safely and effectively provide care to older adults who may test positive for COVID-19. Agencies must also be prepared to rotate out sick providers for extended periods of time and have enough staff to continue to provide care to clients. Similarly, nursing homes need to anticipate that residents and staff will become ill. Clear, comprehensive plans for visitation restrictions, isolation, and notification of family must be devised. Nursing homes staff should be considered front line health care providers. In addition, facilities should have a process in place to transfer residents who may require ED evaluation and criteria to receive them back, if appropriate, following evaluation. Ombudsman programs should develop strategies to continue to perform their vital role safely (The National Long-Term Care Ombudsman Resource Center, 2020).
5. *Hospitals and health care providers have successfully used video-based telehealth to treat COVID-19 patients in their homes and follow up with them after an in-person evaluation* (Hollander & Carr, 2020; Morgan, 2020). Social service agencies that serve elder abuse victims might learn lessons from this success and could consider developing similar systems. Also, there are opportunities for community agencies to collaborate more closely with health care providers and take advantage of the access medical professionals have to at-risk older adults and the ability to view inside their homes through co-assessment via these platforms. Telehealth also requires the development of training programs to ensure that professionals, older adults, and other family members have the requisite skills to use these platforms.
6. *Communities should anticipate that staff who work in organizations that serve victims of elder mistreatment will require significant support during and after this unprecedented pandemic*. Elder mistreatment response is challenging even under normal circumstances, and providers may suffer from secondary traumatic stress (Choi, 2011). This stress can be significantly exacerbated during the COVID-19 pandemic, as workers struggle with vulnerable older adults suffering, their limited ability to assist, and their own personal and family health and economic situations. Support for these workers, frequent check-ins, counseling services, changes in work schedules, and other strategies are critical currently and in the future.

## The Future

Although the immediate future is unclear, eventually life for most New Yorkers will gradually return to some semblance of normalcy. When that transition occurs, we anticipate that many organizations, and the services that they provide, will need to be rebuilt, as will the relationships between agencies and the individual at-risk older adults they serve. It is also likely that older people mistreated during the pandemic will be newly identified and require intensive services. Some features of elder abuse prevention and treatment may have changed permanently, requiring flexibility and adjustment.

Our research and practice collaboration in NYC has already begun and will continue qualitative and quantitative research to formally examine the impact in New York City of this crisis and its aftermath on elder mistreatment victims and the organizations that respond to serve and protect them. We plan to share our results in the future to provide a deeper, more detailed, and more nuanced picture of the consequences, which we hope will inform planning for future crises. We offer initial creative solutions and lessons here, nearly in real time, as we recognize that COVID-19 is already moving to other communities, and we believe that our experiences will help optimize preparation and anticipation of potential challenges.
